# Australia’s continental-scale acoustic tracking database and its automated quality control process

**DOI:** 10.1038/sdata.2017.206

**Published:** 2018-01-30

**Authors:** Xavier Hoenner, Charlie Huveneers, Andre Steckenreuter, Colin Simpfendorfer, Katherine Tattersall, Fabrice Jaine, Natalia Atkins, Russ Babcock, Stephanie Brodie, Jonathan Burgess, Hamish Campbell, Michelle Heupel, Benedicte Pasquer, Roger Proctor, Matthew D. Taylor, Vinay Udyawer, Robert Harcourt

**Affiliations:** 1 Australian Ocean Data Network, Integrated Marine Observing System University of Tasmania, Private Bag 110, Hobart, Tasmania 7001, Australia; 2 School of Biological Sciences, Flinders University, GPO Box 2100, Adelaide, South Australia 5001, Australia; 3 Sydney Institute of Marine Science, Mosman, New South Wales 2088, Australia; 4 Centre for Sustainable Tropical Fisheries and Aquaculture and College of Marine and Environmental Sciences, James Cook University, Townsville, Queensland 4811, Australia; 5 Department of Biological Sciences, Macquarie University, New South Wales 2109, Australia; 6 CSIRO Oceans and Atmosphere, GPO Box 2583, Brisbane 4001, Australia; 7 Ecology and Evolution Research Centre, and School of Biological, Earth and Environmental Sciences, University of New South Wales, Sydney, New South Wales 2052, Australia; 8 Research Institute for Environment and Livelihoods, School of Environment, Charles Darwin University, Northern Territory 0909, Australia; 9 Australian Institute of Marine Science, Townsville, Queensland 4810, Australia; 10 Port Stephens Fisheries Institute, New South Wales Department of Primary Industries, Taylors Beach Rd, Taylors Beach, New South Wales 2316, Australia

**Keywords:** Marine biology, Animal behaviour, Ichthyology, Behavioural ecology, Animal migration

## Abstract

Our ability to predict species responses to environmental changes relies on accurate records of animal movement patterns. Continental-scale acoustic telemetry networks are increasingly being established worldwide, producing large volumes of information-rich geospatial data. During the last decade, the Integrated Marine Observing System’s Animal Tracking Facility (IMOS ATF) established a permanent array of acoustic receivers around Australia. Simultaneously, IMOS developed a centralised national database to foster collaborative research across the user community and quantify individual behaviour across a broad range of taxa. Here we present the database and quality control procedures developed to collate 49.6 million valid detections from 1891 receiving stations. This dataset consists of detections for 3,777 tags deployed on 117 marine species, with distances travelled ranging from a few to thousands of kilometres. Connectivity between regions was only made possible by the joint contribution of IMOS infrastructure and researcher-funded receivers. This dataset constitutes a valuable resource facilitating meta-analysis of animal movement, distributions, and habitat use, and is important for relating species distribution shifts with environmental covariates.

## Background & Summary

Environmental changes affect the distribution and movements of marine species at different spatiotemporal scales^1,2^. Consequently, the long-term monitoring of animal movement is paramount for predicting behavioural responses under changing environmental conditions. Technological advances of animal-borne devices over the past two decades (e.g., radio, acoustic, and satellite transmitters) have revolutionised the field of ethology, enabling ecologists to track a variety of organisms and thereby inform policy makers as to the changing spatiotemporal patterns of species distributions^3^.

**Figure 1:**
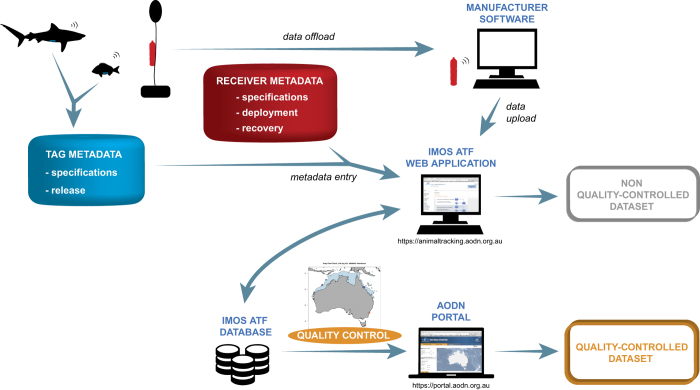
Schematic overview of the IMOS ATF procedure to collect and publish acoustic telemetry data and metadata. Acoustic tags deployed on marine animals are detected when swimming within the detection range of receivers. Researchers offload receiver detections when servicing their equipment and subsequently upload those, along with tag, animal, and receiver metadata into the IMOS ATF web application, where these are available for download in non-quality-controlled format. A quality control procedure is applied on detections of public registered tags and the resulting data are made available through the AODN portal.

Underwater passive acoustic telemetry has become a standard tool for fisheries biologists^4^. A uniquely ID-coded transmitter is attached or implanted in the animal and its high frequency acoustic transmission detected by an array of receivers deployed throughout the animals predicted range (Fig. 1). The detection range of acoustic receivers is typically between 60 and 950 m depending on local geography, bathymetry, and environmental conditions^5^, and this has limited studies to addressing regional (1–50 km) scale hypotheses, and preventing the tracking of migratory species^4,6^. To address this issue, broad-scale integrated networks, composed of acoustic receivers deployed by individual research groups, have become established including in Australia (Integrated Marine Observing System (IMOS)), North America (Atlantic Cooperative Telemetry, California Fish Tracking Consortium, Florida Acoustic Cooperative Telemetry), and South Africa (Acoustic Tracking Array Platform). Many of these networks are co-invested by the global Ocean Tracking Network (OTN)^3,7,8^, and have enhanced collaboration between scientists both nationally and internationally, and facilitated the study of animals moving over broad distances and across management jurisdictions^9^. Any transmitter can be detected on any receiver and the data are fed back to a central repository, thus the network expands the study area of the individual researcher up to the continental scale.

These networks consist of a high number of receivers and transmitters. Over sufficient periods of time, tens of millions of detections are collected across the network, resulting in vast data collections. These datasets are inherently complex, as they simultaneously require receiver and tag equipment specification as well as deployment metadata information. In addition, information infrastructure is often deemed necessary in these collaborative frameworks to facilitate scientific community engagement at such a broad geographical scale. Relational database management systems have been developed to store the resultant large volumes of data along with the creation of online graphical user interfaces for user metadata entry and to enhance data discovery and access^10,11^.

The IMOS monitors coastal waters and open oceans around Australia by deploying observing equipment to address five main research themes: multi-decadal ocean change, climate variability and weather extremes, major boundary currents, continental shelf and coastal processes and marine ecosystem responses^12,13^. The latter theme primarily drives IMOS’ Animal Tracking Facility (ATF), which has deployed acoustic telemetry arrays for over ten years across strategically chosen locations around Australia specifically to facilitate connectivity between independent projects and enable detection of large-scale movements of marine organisms. All observations collected are subsequently made freely available through the Australian Ocean Data Network portal (AODN: https://portal.aodn.org.au/), the primary national repository for marine and climate science data.

Through the IMOS ATF web interface researchers have access to millions of detections from organisms ranging across 117 species tagged throughout Australia. Historical duplicate transmitter IDs and tag transmission collisions caused by multiple transmitters within range of the same receiver or environmental noise^14^ can, however, generate erroneous data thus entailing the development of statistical methods that automatically flag possible invalid detections. Here we present a flexible quality control (QC) procedure for acoustic detection data that assesses, for each individual tag, the validity of detections based on a computed set of metrics. This QC procedure can readily be used for other acoustic telemetry networks. Here we apply this QC algorithm to the raw detections stored in the IMOS ATF back-end database and describe the resulting dataset of detections up until the 11th of April 2017 (Data Citation 1).

## Methods

Passive acoustic telemetry datasets are comprised of three main data groups: detection data, transmitter metadata, and receiver metadata. Transmitter and receiver metadata include equipment specifications and deployment information. For transmitters, this refers to model, type (e.g., pinger versus sensor), transmission interval, information about tagged organisms (e.g., species, size, sex), and tagging location and date. For receivers, this includes model, mooring type, depth, along with deployment and recovery dates and locations.

The IMOS ATF acoustic network is comprised of discrete arrays of acoustic receivers (installations). Depending on the study objectives installations can be configured as curtains, grids, or have no specific formation around features such as reefs or headlands^6^. Acoustic curtains are commonly designed to monitor long-distance migrations or to estimate the fraction of tagged animals that crosses a line of receivers. They may be arranged as ‘gates’ between two headlands or across the entrance of a bay or estuary^15^, or as a cross-shelf curtain^16^. The typical configuration of acoustic curtains consists of individual stations spaced less than 800 m apart to maximise tag detection probability^17^, with the distance between stations determined by a trade-off between average tag detectability and environmental conditions^5^. Receivers can also be configured as grids to estimate home range or residency within specific areas^6^. Spacing between receivers varies from overlapping detection range to infer fine-scale positions^18^ to receivers spaced several kilometres apart to monitor large areas. Each station includes an acoustic receiver either bottom mounted and diver deployable if shallower than 20 m^19^ or on a mooring with an acoustic release for waters 20–150 m deep^5^. The design of the entire IMOS ATF network is based on the acoustic receiver manufacturer’s recommendations (VEMCO Acoustic Gate Design, https://vemco.com/wp-content/uploads/2012/11/gate_design.htm, last accessed 22 Nov 2017) in combination with additional evidence from various scientific studies^5,6,17^.

**Figure 2:**
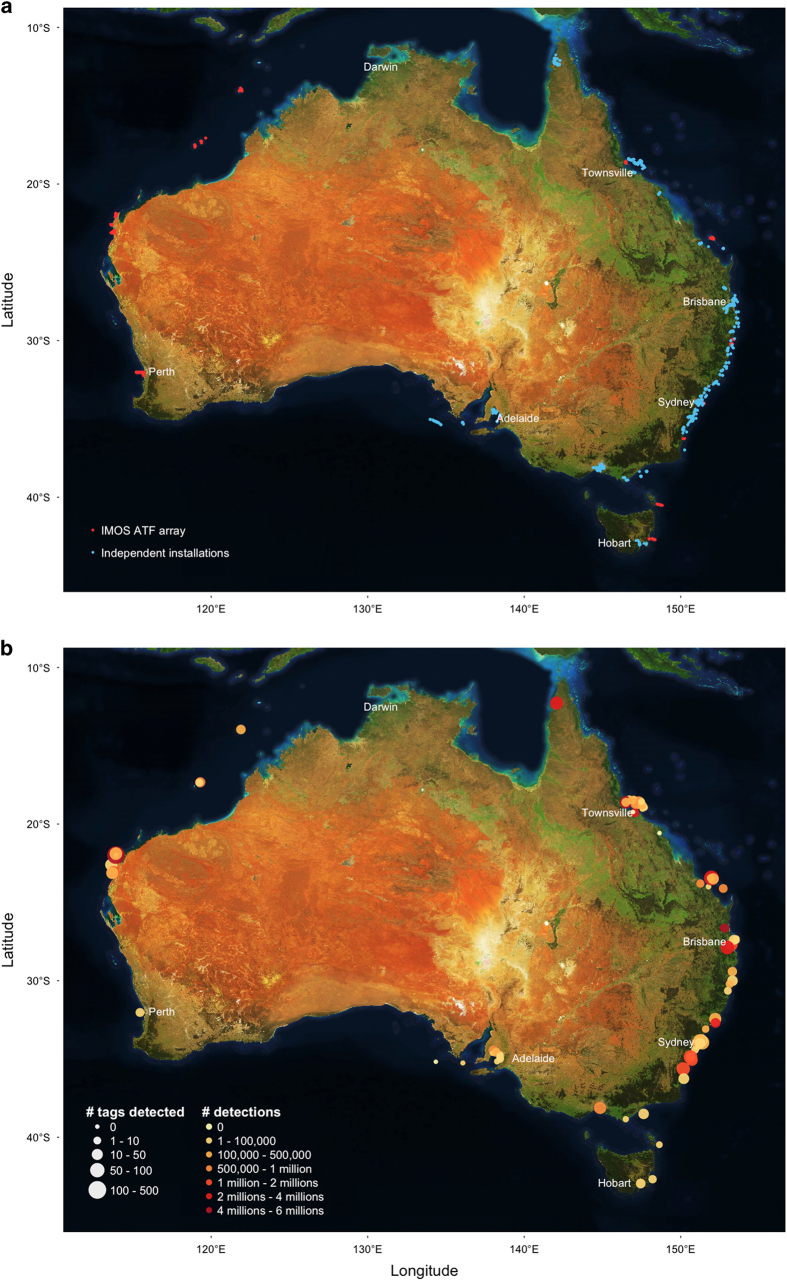
Geographical distribution of acoustic receiver arrays and tag detections around Australia. (**a**) Spatial distribution of IMOS ATF acoustic receiver arrays (red circles) and non-IMOS-funded independent installations (blue circles). Refer to Supplementary Material 1 for a visualisation of the network’s evolution over time. (**b**) Spatial distribution of detections and number of tags detected at each acoustic receiver array. See Supplementary Material 3 for an animation of animal trajectories (*n*=602 individuals) between receiver arrays based on quality-controlled detections for seven species of sharks.

Since 2007 the number of receivers held by IMOS ATF (referred to hereafter as ‘IMOS ATF receiver arrays’) has grown from 70 to 855 (Fig. 2a). In addition, the Australian acoustic telemetry network contains significant co-investment by individual scientists and/or their organisations with 1,305 non-IMOS-funded receivers (referred to as ‘independent installations’) (Fig. 2a and Supplementary Material 1). The data generated by receivers summarises when individual tag IDs were detected, referred to as ‘detections’. Critically, for detection data to be meaningful to the community, independent researchers are encouraged to provide transmitter metadata to the IMOS ATF web application. The database therefore contains detections for tags that have been entered voluntarily in the database (‘registered’ tags and detections) and detections from tags that have not been entered and for which no information is available (‘unregistered’ tags and detections). Acoustic receivers can also record additional data (i.e., animal depth and acceleration, water temperature) from tags equipped with sensors (referred to as ‘sensor tags’). In this case, receivers record a raw integer ranging 0–255, which can subsequently be converted to physical measurements using the sensor’s slope and intercept values following the manufacturers’ standard protocols.

While all data within the ATF database is by default publicly available following IMOS’ open data policy^20^, due to community concerns for data protection during the development of the IMOS ATF web application^21^ two higher levels of data security were created within the database. An *embargo*^22^, in which transmitter and animal metadata are not publicly released for up to three years with the possibility of annual extensions. This has been implemented to facilitate publication by, in particular, early career researchers and students. For *protected data*^23^ access to both tag metadata and detections is restricted. *Protected* status is available only upon application and approval by the IMOS Director and is for projects in which public availability of detections may present an imminent threat to animals or research programs^24^.

The ATF database supports a front-end web application (https://animaltracking.aodn.org.au/) through which users input transmitter and receiver metadata, upload detections from receivers, and may download raw (i.e., not quality controlled) detection data in a CSV file format using filtering tools (Fig. 1). User access to information is managed by this graphical user interface and based on the user’s registration status along with their role within a given project.

### Code availability

All IMOS information infrastructure is open source and thus freely available for others to re-use. The code underlying the IMOS ATF web application and database is accessible through the corresponding GitHub repository (https://github.com/aodn/aatams; Supplementary Material 2). The version controlled R^25^ code used to extract and flag detection data and tag metadata is also available on GitHub (https://github.com/aodn/aatams/tree/master/scripts/R/QC).

## Data Records

### Receiver network

Since IMOS’ inception in 2007, receivers have been deployed at 1891 stations across 103 installations for a total of 7,015 deployments ranging from 113.6° to 154.0°E and 11.8° to 43.1°S (Fig. 2a). IMOS accounts for 40.0% of receivers and 46.9% of all receiver deployments, and represents 30.2 and 21.4% of all stations and installations, respectively.

### Tag network

The IMOS ATF database holds a total of 60.6 million ‘raw’ detections from tags deployed on 117 species (Fig. 2b and Supplementary Material 3). Of these, ‘unregistered’ tags accounted for 8.1 million detections (13.3%) while embargoed and protected data represented 2.9 million detections (4.7%). We herein publish quality-controlled data for 3,777 individual tag deployments totalling 49.6 million detections (Data Citation 1). Of those, 15.1 million (30.5%) and 34.5 million (69.5%) detections occurred on IMOS and non-IMOS installations, respectively.

The quality control procedure generates a separate data file of detections for each tag deployment while tag metadata information is stored in a single file. If the same tag was released multiple times, its detections fall into a separate data record for each deployment. Data and metadata fields are described in Tables 1 and 2.

While raw detection data can be directly downloaded through the IMOS ATF web application (https://animaltracking.aodn.org.au) the quality-controlled dataset is available through the AODN portal (https://portal.aodn.org.au). This dataset has been assigned a DOI and may be directly accessed using the following URL: https://portal.aodn.org.au/search?uuid=0ede6b3d-8635-472f-b91c-56a758b4e091. Alternatively, individual data files may be downloaded through the AODN S3 browser (http://data.aodn.org.au/?prefix=IMOS/AATAMS/acoustic_tagging). This static dataset will be complemented by a dynamic quality-controlled dataset that will be updated annually.

## Technical Validation

Using a back-up of the IMOS ATF database we extracted detection data and metadata for every registered transmitter, aggregating sensor and detection data for dual sensor tags. To identify ‘false detections’ caused by transmission collisions we computed the number of times each transmitter had been detected at each installation, along with time intervals between consecutive detections. If, at a given installation, a tag was only detected once or if there were more long (>12 h) than short periods (<30 min) between detections^26^, then the corresponding detections were flagged as ‘likely invalid’ (‘FDA_QC’ flag=2) (Table 1).

We then tested the validity of individual detections against movement metrics by computing the distance and swim speed between consecutive detections. We first obtained a high-resolution map of Australia through the ‘rworldmap’ and ‘rworldxtra’ libraries^27^ (https://cran.r-project.org/web/packages/rworldxtra/index.html, last accessed 22 Nov 2017) which we then rasterised and transformed into a transition object. All raster cells off the Australian landmass were assigned a given numerical value thus enabling to identify subsequently any location on land. To reduce computation requirements we identified all receivers onto which a given transmitter was detected prior to compiling unique trips between those stations. We then generated 200 locations through linear interpolation for each trip between two stations to determine when straight-line trajectories involved movement over land. When all those interpolated points were located in the water or when two consecutive detections occurred on the same river installation, we computed the straight-line distance between receivers. Conversely, whenever any interpolated location was detected on land, we calculated the computationally intensive ‘least-cost’ distance between those two stations using the costDistance function in the ‘gdistance’ R package (https://cran.r-project.org/web/packages/gdistance/index.html, last accessed 22 Nov 2017) which accounted for the shape of Australia’s coastline. While the spatial resolution of our map was appropriate for representing the ocean’s coastline it was too coarse for small river systems. Thus, for consecutive detections occurring in two different rivers (or in a river and the adjacent ocean), in addition to the above described distance calculation, our algorithm also systematically computed the straight-line distance between the river receiver and the closest point on the coastline. A given detection was then flagged as ‘invalid’ if both the distances with the previous and next receiver were greater than 1,000 km (‘Distance_QC’=2) or if the corresponding travel velocities were greater than 10 m.s^−1^ (‘Velocity_QC’=2) (Table 1). The 1,000 km distance threshold is a conservative value based on the greatest minimum distance between neighbouring installations (mean±s.d.=61.3±148.6 km, range=0.3–1,005 km, *n*=104 installations), while the velocity threshold was assigned from maximum swim speeds recorded for southern bluefin tuna (*Thunnus maccoyii*, Castelnau, 1872) and mako shark (*Isurus oxyrinchus*, Rafinesque, 1810), the two fastest species in the IMOS ATF database^28,29^. Note that for detections occurring at the same time on two distinct receivers we approximated to one second the corresponding time interval to be able to compute swim speed.

We also tested the validity of individual detections from an ecological standpoint by comparing detection locations against each species known distribution. For each tagged species in the IMOS ATF database, we downloaded a shapefile representing its geographical distribution area from the Atlas of Living Australia (http://www.ala.org.au/) based on the Australian National Fish Expert Distributions^30^. Detections failed this test if they occurred outside of a species’ known occurrence area (‘DetectionDistribution_QC’=2), allowing for uncertainties in compiled distributions and climate-induced species range shifts by extending the original area’s latitudinal range by 500 km (refs 31,32) (Table 1). Due to historical duplicate transmitter IDs and missing species distributions for marine invertebrates we introduced a complementary method to test the geographical distribution of detections by calculating the distance from each detection to the tag deployment location. A given detection passed this test (‘DistanceRelease_QC’=1) if it occurred within a 500 km radius of where the tagged animal was released, a conservative threshold value best suited for relatively resident species (Table 1).

Finally, we also isolated (1) detections occurring before a tag’s release date ('ReleaseDate_QC’=2) allowing for potential time zone discrepancies, and (2) likely invalid release locations (‘ReleaseLocation’=2) by calculating the straight-line distance with the first detection and testing whether the release coordinates were within the ALA species distribution area (Table 1).

For researchers to re-use detection data easily we computed an additional field, ‘Detection_QC’, summarising the output of the five first tests undertaken on individual detections, i.e., ‘FDA_QC’, ‘Distance_QC’, ‘Velocity_QC’, ‘DetectionDistribution_QC’, and ‘DistanceRelease_QC’ (Table 1). If five of these fields had a valid QC flag of 1, then ‘Detection_QC’ was assigned 1, meaning the detection is deemed ‘valid’. If only four of these fields had a QC flag of 1 then ‘Detection_QC’=2, meaning the detection is ‘likely to be valid’. Detections having three or less than three of these five fields with a QC flag of 1 were considered ‘likely invalid’ or ‘invalid’, respectively.

43.2 million detections (87.1%) were flagged by the quality control process as valid (‘Detection_QC’=1) while 48.9 millions (98.6%) were valid or likely valid (‘Detection_QC’=1 or 2). As a result, about 730,000 detections (1.4%) were identified as invalid or likely invalid (‘Detection_QC’=3 or 4), with 126 tags (3.3%) having all their detections flagged as such, primarily because of potentially inaccurate or missing species distribution areas.

**Figure 3:**
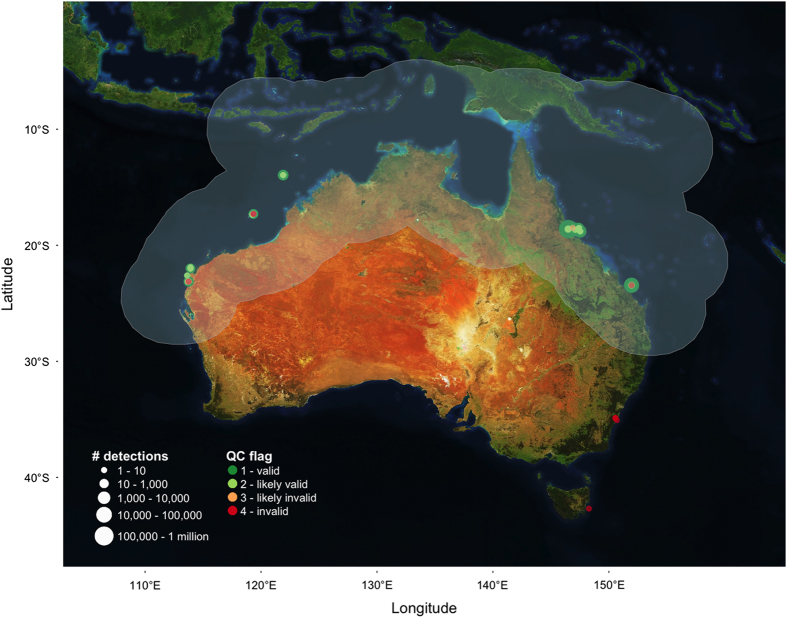
Spatial distribution of detections of tagged grey reef sharks colour-coded based on their likely validity. Example of species map produced as part of the technical validation showing the spatial distribution of detections for tags deployed on grey reef sharks (*Carcharhinus amblyrhynchos*, Bleeker, 1856). Valid detections (‘Detection_QC’ <=2) are indicated by green circles, likely invalid and invalid detections by orange and red circles respectively. The Atlas of Living Australia’s species distribution area with the added 500 km buffer area is represented in shaded light blue.

In addition to the scripts for downloading and analysing detection data, we produced multiple log files providing diagnostic information about each tag and a summary of their QC flags. Such validation was essential to identify metadata content issues and was an invaluable tool to strengthen the robustness of both the IMOS ATF web application and underlying database. Furthermore, for each of the 117 species detected on the Australian acoustic receiver network, we plotted the location of valid and invalid detections (Fig. 3), thus enabling us to visualise how the QC algorithm performed with changing parameters and threshold values. Species occurrence data validated through the present approach will subsequently be shared with (1) the Atlas of Living Australia, and (2) FishMap experts (http://fish.ala.org.au/) to contribute to existing biodiversity records and thus help refine geographical distribution maps.

## Usage Notes

**Figure 4:**
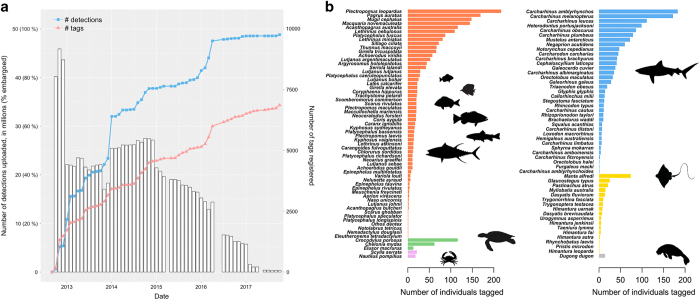
Cumulative number of tags registered and detections uploaded on the IMOS ATF web application through time along with number of animals tagged for each species. (**a**) Cumulative number of tags registered and detections uploaded on the IMOS ATF web application. The proportion of detections embargoed is overlaid on this graph and represented by white histogram bars. (**b**) Inventory of the number of animals tracked for each of the 117 species and for which detections are available in the data descriptor.

Since the launch of the IMOS ATF web application in 2012 the number of detections uploaded, along with the number of tags registered by users and species tracked has grown steadily (Fig. 4a,b). During this period the number of tags embargoed or protected and their corresponding number of detections has decreased drastically so that today only a minority of tags are associated with any security measures (Fig. 4a).

The primary point of access to the quality-controlled detections is through the AODN portal (Data Citation 1) where users can visualise species detection occurrences and subset data using a set of filters, e.g., species name, transmitter ID, tag project name, installation name. Detection data, along with the corresponding tag metadata can then be downloaded in CSV format through this portal. Alternatively, all data files and a master tag metadata file are also available through the AODN S3 browser (http://data.aodn.org.au/?prefix=IMOS/AATAMS/acoustic_tagging). Data file names use the following convention, whose combination of IDs may be subsequently used to identify the corresponding metadata record in the tag metadata file: ‘TransmitterID_TagID_ReleaseID.csv’ (Table 2). Note that a given tag ID may be associated with multiple releases in case of capture/re-deployment and that multiple transmitter IDs may be associated with the same tag ID for dual sensor tags. Based on our technical validation process we recommend users discard all detections flagged as invalid or likely invalid (‘Detection_QC’=3 or 4). Although some verification may be required, working with valid and likely valid detections (‘Detection_QC’=1 and 2) provides the most accurate picture of individual movement while retaining significantly more data than valid detections only (‘Detection_QC’=1) (Supplementary Material 4).

## Additional information

**How to cite this article:** Hoenner, X. *et al.* Australia’s continental-scale acoustic tracking database and its automated quality control process. *Sci. Data* 5:180206 doi: 10.1038/sdata.2017.206 (2018).

**Publisher’s note:** Springer Nature remains neutral with regard to jurisdictional claims in published maps and institutional affiliations.

## Supplementary Material



Supplementary Materials

Supplementary Movie 1

Supplementary Movie 2

## Figures and Tables

**Table 1:** Name, description, and values of each QC detection data field.

**Data field**	**Description**	**Values or units**
*transmitter_id*	Combination of code map and ping ID. Dual sensor tags are associated with multiple transmitter IDs.	Alphanumeric sequence, e.g., A69-9002-12345
*installation_name*	Name of installation on which the transmitter was detected. An installation typically consists of multiple receiving stations.	
*station_name*	Name of receiving station on which the transmitter was detected. Acoustic receivers typically get deployed multiple times at the same station.	
*receiver_name*	Name of acoustic receiver; combines receiver model with its serial number.	Alphanumeric sequence, e.g., VR2W-123456
*detection_timestamp*	Date and time of tag detection.	Year-Month-Day Hour:Minute:Second
*longitude*	Longitude at which receiver was deployed and tag was detected.	In decimal degrees
*latitude*	Latitude at which receiver was deployed and tag was detected.	In decimal degrees
*sensor_value*	Physical measurement recorded by a tag’s sensor, if applicable.	If sensor data has not been converted then sensor_unit=‘ADC’ and values range from 0 to 255
*sensor_unit*	Physical unit associated with sensor values.	Either ‘ADC’, ‘°C’, ‘m’ or ‘m/s^2^’
*FDA_QC*	Quality control flag for the false detection algorithm.	1: passed2: failed
*Velocity_QC*	Velocity from previous and next detections both≤10 m.s^−1^?	1: yes2: no
*Distance_QC*	Distance from previous and next detections both≤1000 km?	1: yes2: no
*DetectionDistribution_QC*	Detection occurred within expert distribution area?	1: yes2: no3: test not performed
*DistanceRelease_QC*	Detection occurred within 500 km of release location?	1: yes2: no
*ReleaseDate_QC*	Detection occurred before tag release date?	1: yes2: no
*ReleaseLocation_QC*	Tag release geographical coordinates within expert distribution area and/or within 500 km of first detection?	1: yes2: no3: test not performed
*Detection_QC*	Composite detection flag indicating likely validity of detections.	1: valid detection2: probably valid detection3: probably invalid detection4: invalid detection

**Table 2:** Name, description, and values of each metadata field.

**Metadata field**	**Description**	**Values or units**
*transmitter_id*	Combination of code map and ping ID.	Alphanumeric sequence, e.g., A69-9002-12345
*tag_id*	Unique tag ID. Dual sensor tags have different transmitter IDs but the same tag ID.	
*release_id*	Unique tag release ID. A given tag ID may be associated with several release IDs if it has been re-deployed.	
*tag_project_name*	Project name under which a tag was registered.	
*scientific_name*	Tagged species scientific name.	
*common_name*	Tagged species common name.	
*release_longitude*	Longitude at which tag was deployed.	In decimal degrees
*release_latitude*	Latitude at which tag was deployed.	In decimal degrees
*ReleaseDate*	Date and time at which tag was deployed.	Year-Month-Day Hour:Minute:Second
*sensor_slope*	Slope used in the linear equation to convert raw sensor measurements.	
*sensor_intercept*	Intercept used in the linear equation to convert raw sensor measurements.	
*sensor_type*	Type of sensor.	i.e., pinger, temperature, pressure, accelerometer
*sensor_unit*	Sensor unit.	
*tag_model_name*	Tag model.	
*tag_serial_number*	Tag serial number.	
*tag_expected_life_time_days*	Tag expected life time.	In days
*tag_status*	Tag status.	e.g., deployed, lost
*sex*	Animal sex.	
*measurement*	Animal measurements.	
*dual_sensor_tag*	Is the tag a dual sensor tag?	
