# Supplementary material for: Australia’s continental-scale acoustic tracking database and its automated quality control process
Source: Sci Data. 2018 Jan 30;5:170206. doi: 10.1038/sdata.2017.206 (PMC5789868; doi:10.1038/sdata.2017.206)
Supplement: Supplementary Materials [file sdata2017206-s2.pdf]

Supplementary materials

Supplementary material 2: page 2

Supplementary material 3: page 3 – 7

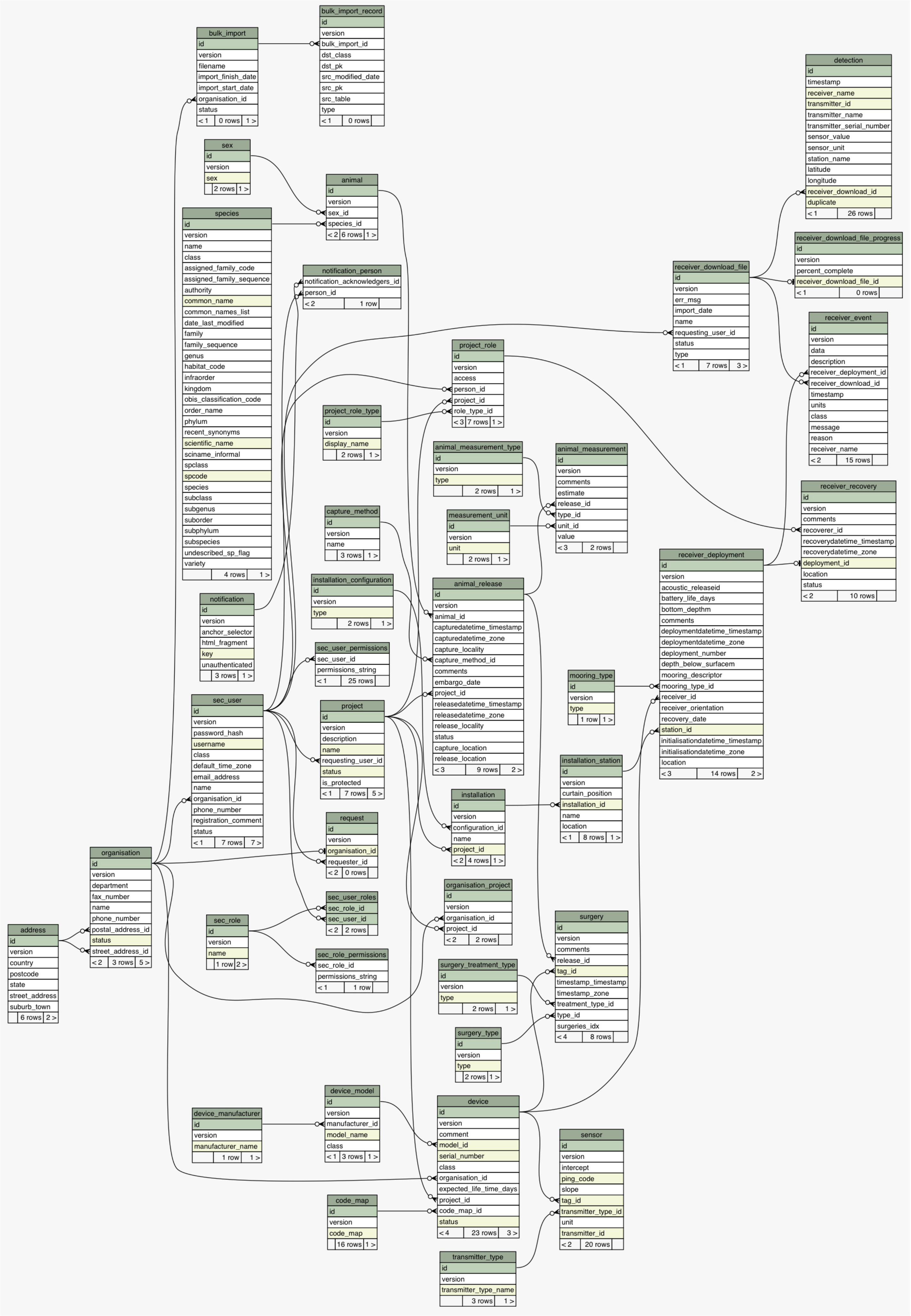

Supplementary material 2: Schematic representing the IMOS ATF acoustic telemetry database structure, relationships, and fields.

Animal Tracking - Acoustic Tagging - data currently available via the portal

Data summary - Detections by species - report run on Tuesday 31 October 2017

IMOS Integrated Marine Observing System

**# transmitters:**	Number of transmitters deployed.
**# releases:**	Number of animal releases.
**# embargoed releases:**	Number of embargoed releases.
**# releases with detections:**	Number of released animals that have been detected.
**# releases with location:**	Number of animal releases for which capture/release latitude and longitude coordinates are available.
**# protected:**	Number of transmitters that are part of a protected project.
**# detections:**	Total number of detections.
**# public detections:**	Total number of public detections.
**First detection:**	Data at which the first tag detection occurred (format: dd/mm/yyyy).
**Last detection:**	Date at which the last tag detection occurred (format: dd/mm/yyyy).
**# days of data (range):**	Number of days between the first and last detection for each animal released (minimum - maximum).
**Animal Tracking Facility:**	http://imos.org.au/animaltracking.html: web app: https://animaltracking.aodn.org.au/.

Common name	# transmitters	# releases	# embargoed releases	# releases with location	# protected	Latest embargo date	# detections	# public detections	First detection	Last detection	# days of data (range)
**Registered tags**											
[a horn shark]	2	2	0	2	0	null	0	0	null	null	null
All range test tags	198	2	0	2	0	null	11,236,123	11,236,123	02/12/2007	03/10/2017	0.0 - 3062.0
All registered tags with no species info	744	38	0	38	20	null	3,124,803	3,124,803	07/12/2007	10/07/2017	0.0 - 2937.0
[a threadfin salmon]	3	3	0	3	0	null	0	0	null	null	null
Australian Bass	224	220	0	220	0	null	2,094,585	2,094,585	10/01/2008	13/08/2016	0.0 - 2628.0
Australian Lungfish	20	20	0	20	0	null	2,103,594	2,103,594	25/06/2013	23/10/2015	2.0 - 846.0
Australian Sharpnose Shark	39	39	0	39	0	null	737	737	29/10/2011	20/11/2013	0.0 - 599.0
[a wedgefish]	1	1	0	1	0	null	11,492	11,492	30/08/2009	29/12/2012	1216.0 - 1216.0
Barcheek Coral Trout	30	30	0	30	0	null	418,913	418,913	23/11/2010	08/03/2015	0.0 - 374.0
Barramundi	222	222	0	222	0	null	4,119,627	4,119,627	16/09/2010	19/05/2016	0.0 - 770.0
Bigeye Snapper	29	29	0	29	0	null	3,536,500	3,536,500	10/12/2009	09/05/2012	7.0 - 708.0
Black Bream	2	2	0	2	0	null	3,051	3,051	29/11/2009	19/12/2011	0.0 - 749.0
Blackspotted Whipray	2	2	0	2	0	null	206,752	206,752	13/11/2009	01/05/2011	4.0 - 533.0
Blacktip Reef Shark	190	189	0	189	0	null	3,756,341	3,756,341	18/11/2007	26/05/2017	0.0 - 2258.0
Blind Shark	16	16	0	16	0	null	240	240	16/02/2012	19/09/2016	0.0 - 1362.0
Bluebarred Parrotfish	1	1	0	1	0	null	12	12	26/05/2009	07/06/2009	11.0 - 11.0
Blue Catfish	18	18	0	18	0	null	623,610	623,610	19/04/2012	12/01/2017	0.0 - 438.0
Bluelined Rabbitfish	33	33	33	33	0	08/12/2018	0	0	null	null	null

Supplementary material 3: Table summarising the detection status for individual species registered in the IMOS ATF acoustic telemetry database as of October 2017. These summary statistics are generated on a monthly basis and available by clicking on the 'Animal Tracking - Acoustic Tracking (Species)' link at the following URL: http://imos.org.au/facilities/datareports/ (last accessed 9 Nov 2017)

Common name	# transmitters	# releases	# embargoed releases	# releases with location	# protected	Latest embargo date	# detections	# public detections	First detection	Last detection	# days of data (range)
Bluespine Unicornfish	3	3	0	3	0	null	417	417	30/01/2009	06/11/2009	11.0 - 280.0
Bluespotted Coral Trout	17	17	0	17	0	null	54,795	54,795	13/01/2012	18/10/2014	0.0 - 374.0
Bluespotted Fantail Ray	5	5	0	5	0	null	123,358	123,358	21/08/2009	04/05/2011	0.0 - 533.0
Bluespotted Flathead	96	96	16	96	0	15/05/2018	81,940	81,645	14/08/2013	30/07/2016	0.0 - 392.0
Blue Threadfin	8	8	0	8	0	null	9	9	25/11/2010	27/11/2010	2.0 - 2.0
Bluethroat Wrasse	5	5	0	5	0	null	4	4	05/11/2009	06/11/2009	1.0 - 1.0
Brassy Drummer	14	14	0	14	0	null	218,296	218,296	19/09/2011	20/03/2013	18.0 - 546.0
Broadnose Shark	129	129	31	129	0	25/04/2018	76,414	75,866	10/12/2007	01/05/2017	0.0 - 1659.0
Bronze Whaler	54	54	0	54	0	null	289,738	289,738	28/12/2009	31/01/2016	0.0 - 1452.0
Bull Shark	197	195	0	195	0	null	1,231,224	1,231,224	04/04/2009	13/05/2017	0.0 - 2662.0
Chinaman Rockcod	5	5	0	5	0	null	206	206	04/12/2007	01/11/2009	0.0 - 190.0
Cobia	1	1	0	1	0	null	0	0	null	null	null
Common Blacktip Shark	15	15	0	15	0	null	71,062	71,062	09/06/2011	02/08/2016	0.0 - 1360.0
Common Coral Trout	288	288	0	288	0	null	1,469,469	1,469,469	28/05/2008	20/03/2016	0.0 - 1180.0
Common Stingaree	11	11	0	11	0	null	7,531	7,531	22/11/2012	27/05/2013	1.0 - 62.0
Cowtail Stingray	25	25	0	25	0	null	105,726	105,726	22/02/2008	21/03/2013	0.0 - 1293.0
Creek Whaler	20	20	0	20	0	null	30	30	15/10/2012	16/10/2012	0.0 - 1.0
Devil Ray	130	130	35	130	0	05/02/2020	602,191	601,988	23/11/2007	26/05/2017	0.0 - 2398.0
Draughtboard Shark	43	43	0	43	0	null	2,423	2,423	30/03/2015	20/06/2015	0.0 - 80.0
dugong	18	18	0	18	0	null	23,672	23,672	25/07/2012	12/09/2015	0.0 - 1113.0
Dusky Flathead	106	106	0	106	0	null	740,542	740,542	25/11/2009	13/06/2016	0.0 - 1277.0
Dusky Whaler	128	128	0	128	0	null	20,115	20,115	20/03/2010	07/12/2016	0.0 - 1631.0
Eastern Blue Groper	107	107	0	107	0	null	585,289	585,289	10/01/2008	13/08/2016	33.0 - 2622.0
eastern fiddler ray	50	50	37	50	0	07/04/2020	209,730	53,500	28/06/2013	23/06/2017	0.0 - 602.0
elegant seasnake	6	6	0	6	0	null	0	0	null	null	null
Elephantfish	21	21	0	21	0	null	9,090	9,090	07/11/2008	19/06/2017	16.0 - 1561.0
emperor nautilus	21	21	0	21	0	null	144,877	144,877	21/09/2008	24/06/2014	32.0 - 1760.0
Estuary Stingray	14	14	0	14	0	null	26,104	26,104	18/01/2013	18/08/2014	30.0 - 519.0
Fossil Shark	1	1	0	1	0	null	0	0	null	null	null
Freshwater Sawfish	1	1	0	1	0	null	20,449	20,449	02/12/2012	30/05/2014	543.0 - 543.0
Freshwater Whipray	9	9	0	9	0	null	21,591	21,591	15/10/2015	18/04/2016	98.0 - 160.0
giant mud crab	35	29	0	29	0	null	131,515	131,515	03/01/2009	27/08/2016	0.0 - 2792.0
Giant Shovelnose Ray	31	31	0	31	0	null	6,321	6,321	24/02/2008	08/10/2015	0.0 - 622.0
Giant Trevally	35	35	0	35	0	null	20,016	20,016	08/05/2012	08/01/2015	0.0 - 373.0
Golden Snapper	2	2	0	2	0	null	179,939	179,939	16/09/2010	31/10/2012	605.0 - 702.0
Golden Trevally	10	10	0	10	0	null	0	0	null	null	null
Graceful Shark	1	1	0	1	0	null	4	4	09/09/2010	09/09/2010	0.0 - 0.0
Greasy Rockcod	7	7	0	7	0	null	863	863	29/05/2008	04/11/2009	0.0 - 155.0
Great Hammerhead	7	7	0	7	0	null	22,465	22,465	17/03/2012	05/09/2014	0.0 - 798.0

Common name	# transmitters	# releases	# embargoed releases	# releases with location	# protected	Latest embargo date	# detections	# public detections	First detection	Last detection	# days of data (range)
Greenfin Parrotfish	14	14	0	14	0	null	1,472	1,472	03/06/2008	09/11/2009	0.0 - 168.0
Green Jobfish	3	3	0	3	0	null	2,741	2,741	18/01/2009	24/05/2011	10.0 - 101.0
green turtle	132	132	0	132	0	null	2,686,993	2,686,993	15/01/2011	28/11/2015	0.0 - 607.0
Grey Morwong	1	1	0	1	0	null	13,130	13,130	22/08/2013	11/01/2014	141.0 - 141.0
Greynurse Shark	21	21	10	21	0	15/11/2018	1,151	549	20/08/2012	07/01/2016	0.0 - 160.0
Grey Reef Shark	236	236	0	236	0	null	5,171,171	5,171,171	23/11/2007	26/05/2017	0.0 - 2511.0
Gummy Shark	87	86	0	86	0	null	291,149	291,149	18/02/2008	07/06/2016	0.0 - 2428.0
Harlequin Fish	10	10	0	10	0	null	1	1	16/03/2011	16/03/2011	0.0 - 0.0
Jenkins' Whipray	5	5	0	5	0	null	36,109	36,109	31/08/2009	31/10/2012	67.0 - 1156.0
Jungle Perch	16	16	0	16	0	null	0	0	null	null	null
Lemon Shark	76	75	1	75	0	15/06/2019	1,005,548	1,000,237	19/11/2007	24/11/2016	0.0 - 1473.0
Leopard Whipray	1	1	0	1	0	null	5	5	17/01/2011	21/02/2012	399.0 - 399.0
Longnose Parrotfish	1	1	0	1	0	null	0	0	null	null	null
Longspine Flathead	10	10	0	10	0	null	925	925	29/08/2013	04/02/2014	159.0 - 159.0
Luderick	104	104	17	104	0	16/02/2020	226,574	204,796	03/02/2012	27/05/2017	0.0 - 763.0
Mahi Mahi	70	70	1	70	0	30/01/2019	101,512	101,511	22/12/2011	24/03/2013	0.0 - 404.0
Mangrove Jack	79	79	0	79	0	null	1,116,090	1,116,090	16/09/2010	20/02/2014	0.0 - 780.0
Mary River turtle	13	13	0	13	0	null	724,274	724,274	21/06/2012	23/10/2015	407.0 - 771.0
Mulloway	105	105	17	105	0	31/03/2019	1,052,559	1,024,060	26/10/2009	14/04/2017	0.0 - 1449.0
Nervous Shark	12	12	0	12	0	null	17,953	17,953	23/02/2008	27/07/2012	2.0 - 1616.0
Ocean Jacket	7	7	0	7	0	null	39	39	26/07/2013	01/09/2013	0.0 - 36.0
Pigeye Shark	12	12	0	12	0	null	333	333	26/12/2010	30/01/2014	0.0 - 483.0
Pinkeye Mullet	22	22	0	22	0	null	370,508	370,508	23/04/2013	02/03/2015	0.0 - 596.0
Pink Whipray	4	4	0	4	0	null	276,222	276,222	26/06/2009	15/03/2013	0.0 - 1289.0
Porcupine Ray	6	6	0	6	0	null	3,624	3,624	25/02/2008	08/01/2012	58.0 - 786.0
Port Jackson Shark	157	157	0	157	0	null	1,619,741	1,619,741	22/08/2010	28/09/2017	0.0 - 1978.0
Rankin Cod	9	9	0	9	0	null	15,195	15,195	27/05/2008	09/03/2013	0.0 - 1530.0
Red Bass	52	52	0	52	0	null	45,491	45,491	24/03/2015	06/09/2016	0.0 - 518.0
Redblotched Wrasse	20	20	0	20	0	null	115,319	115,319	02/12/2007	11/11/2011	47.0 - 679.0
Red Emperor	30	30	0	30	0	null	618	618	19/03/2011	25/07/2013	0.0 - 296.0
Redthroat Emperor	109	109	0	109	0	null	311,119	311,119	31/03/2011	02/04/2015	0.0 - 374.0
Reticulate Whipray	8	8	0	8	0	null	61,531	61,531	13/11/2008	22/08/2011	15.0 - 1012.0
Rock Blackfish	43	43	0	43	0	null	18,746	18,746	02/12/2011	27/11/2012	0.0 - 334.0
saltwater crocodile	225	222	0	222	0	null	2,089,803	2,089,803	15/08/2008	03/08/2016	0.0 - 2428.0
Sandbar Shark	132	132	0	132	0	null	213,209	213,209	29/06/2011	06/05/2017	0.0 - 1984.0
sand flathead (mixed)	29	26	0	26	0	null	30,446	30,446	05/03/2008	16/04/2015	0.0 - 2228.0
Sand Whiting	90	90	0	90	0	null	944,154	944,154	25/11/2009	19/07/2013	0.0 - 916.0
sawfishes	1	1	0	1	0	null	190	190	31/10/2015	15/11/2015	15.0 - 15.0
School Shark	76	70	0	70	0	null	157,544	157,544	25/02/2008	27/08/2016	0.0 - 3085.0

Common name	# transmitters	# releases	# embargoed releases	# releases with location	# protected	Latest embargo date	# detections	# public detections	First detection	Last detection	# days of data (range)
Sea Mullet	182	182	20	182	0	23/03/2019	1,146,946	1,146,669	02/07/2011	07/09/2016	0.0 - 1181.0
Shark spp.	12	12	9	12	0	01/11/2019	9,369	56	10/12/2012	18/08/2017	61.0 - 1696.0
Silver Drummer	19	19	0	19	0	null	368,799	368,799	22/01/2009	09/01/2013	0.0 - 1448.0
Silvertip Shark	52	50	0	50	0	null	238,284	238,284	28/12/2007	09/12/2015	0.0 - 1471.0
Silver Trevally	6	6	6	6	0	08/12/2018	12,660	0	20/07/2015	02/09/2016	11.0 - 376.0
Sixspine Leatherjacket	5	5	0	5	0	null	680	680	28/11/2012	14/12/2013	13.0 - 58.0
Sliteye Shark	7	7	0	7	0	null	7,108	7,108	26/07/2012	16/10/2014	0.0 - 756.0
Smooth Stingray	38	38	12	38	0	16/03/2018	63,827	309	25/03/2011	19/05/2017	2.0 - 1455.0
Snapper	246	246	0	246	0	null	1,614,642	1,614,642	17/10/2009	22/07/2014	0.0 - 900.0
Southern Bluefin Tuna	1000	996	0	996	0	null	155,491	155,491	16/06/2009	28/12/2016	0.0 - 1893.0
Southern Bluespotted Flathead	1	1	0	1	0	null	7	7	01/04/2015	01/04/2015	0.0 - 0.0
Southern Eagle Ray	28	22	0	22	0	null	873,154	873,154	06/11/2008	30/03/2017	0.0 - 2937.0
Southern Sand Flathead	27	27	0	27	0	null	1,770	1,770	22/12/2009	20/04/2015	0.0 - 1643.0
Southern Sawshark	2	2	0	2	0	null	0	0	null	null	null
spangled emperor	16	16	0	16	0	null	74,000	74,000	19/08/2013	08/09/2014	0.0 - 374.0
Spangled Emperor	130	130	0	130	0	null	1,113,720	1,113,720	01/12/2007	07/01/2016	0.0 - 2817.0
Spanish Mackerel	30	30	0	30	0	null	5,074	5,074	03/08/2013	14/04/2014	0.0 - 115.0
speartooth shark	26	26	0	26	0	null	41,456	41,456	27/11/2012	09/01/2016	0.0 - 781.0
spine-bellied seasnake	19	19	0	19	0	null	0	0	null	null	null
Spotted Wobbegong	32	32	0	32	0	null	2,140,477	2,140,477	18/06/2008	14/04/2014	2.0 - 1799.0
Surf Parrotfish	21	21	0	21	0	null	111,400	111,400	23/05/2009	02/12/2012	4.0 - 521.0
Temperate Bass	24	24	0	24	0	null	3,125,458	3,125,458	19/06/2012	05/10/2015	0.0 - 898.0
Tiger Flathead	11	11	0	11	0	null	24,470	24,470	01/04/2015	19/06/2015	0.0 - 78.0
Tiger Shark	45	45	0	45	0	null	107,415	107,415	26/02/2008	07/12/2016	0.0 - 1484.0
Turrum	16	16	0	16	0	null	121,598	121,598	01/12/2007	04/07/2011	2.0 - 1309.0
Warrior Catfish	22	22	0	22	0	null	9,753	9,753	13/05/2015	26/05/2016	1.0 - 379.0
Weasel Shark	6	6	0	6	0	null	13,744	13,744	22/08/2011	27/02/2014	0.0 - 535.0
Western Blue Groper	15	15	0	15	0	null	59,094	59,094	20/10/2009	07/05/2010	0.0 - 199.0
Western Yellowfin Bream	1	1	0	1	0	null	0	0	null	null	null
Whaler Shark	25	25	0	25	0	null	25,999	25,999	14/12/2011	19/09/2016	0.0 - 462.0
Whale Shark	29	29	0	29	0	null	1,991	1,991	23/08/2010	10/11/2013	21.0 - 534.0
Whiskery Shark	1	1	0	1	0	null	11	11	18/09/2014	23/10/2014	35.0 - 35.0
White Shark	466	466	134	466	176	31/12/2020	445,915	122,482	12/01/2008	23/09/2017	0.0 - 2178.0
Whitespotted Dogfish	11	11	0	11	0	null	1,747	1,747	28/04/2008	11/05/2011	0.0 - 890.0
Whitetip Reef Shark	26	26	0	26	0	null	360,893	360,893	31/12/2008	23/05/2017	83.0 - 1638.0
Winghead Shark	1	1	0	1	0	null	0	0	null	null	null
Wobbegong	1	1	0	1	0	null	1,159	1,159	18/06/2008	03/01/2010	564.0 - 564.0
Yellowedge Coronation Trout	8	8	0	8	0	null	3,886	3,886	07/01/2008	16/10/2012	0.0 - 1347.0
Yellowfin Bream	163	163	1	163	0	30/12/2017	1,841,471	1,836,946	13/06/2009	07/10/2016	0.0 - 1636.0

Common name	# transmitters	# releases	# embargoed releases	# releases with location	# protected	Latest embargo date	# detections	# public detections	First detection	Last detection	# days of data (range)
Yellowtail Emperor	37	37	0	37	0	null	42,323	42,323	30/01/2009	09/11/2009	59.0 - 240.0
Yellowtail Kingfish	112	112	0	112	0	null	191,953	191,953	04/11/2010	21/04/2014	0.0 - 1247.0
Zebra Shark	18	18	0	18	0	null	11,884	11,884	12/02/2012	13/02/2017	54.0 - 1212.0

Unregistered tags

All unregistered tags	6169	0	0	0	0	null	6,880,547	6,880,547	29/11/2007	11/06/2017	0.0 - 2827.0

	Total number of releses/detections	Number of releases/detections that are public	Number of releases/detections currently embargoed	Currently embargoed for 1 year	Currently embargoed for 2 years	Currently embargoed for 3 years	Currently embargoed for more than 3 years
**Species**	136	120	16	12	9	4	1
**Animals**	7,474	7,094	380	120	185	39	1
**Tags**	14,584	14,204	380	123	196	39	1
**Detections**	77,707,159	77,079,966	627,193	92,259	460,744	18,047	8,136

	Number of times detected	Number of tags	Number of detections
**Registered tags:**	0	2,992	0
	1	132	132
	>1	4,667	67,233,938
	Subtotal	7,791	67,234,070
**Unregistered tags:**	1	3,328	3,328
	>1	2,841	6,877,219
	Subtotal	6,169	6,880,547
	**Total**	13,960	74,114,617
